# Opuntia Fruits as Food Enriching Ingredient, the First Step towards New Functional Food Products

**DOI:** 10.3390/molecules25040916

**Published:** 2020-02-18

**Authors:** Anna Oniszczuk, Agnieszka Wójtowicz, Tomasz Oniszczuk, Arkadiusz Matwijczuk, Ahlem Dib, Ewa Markut-Miotła

**Affiliations:** 1 Department of Inorganic Chemistry, Medical University of Lublin, Chodźki 4a, 20-093 Lublin, Poland; 2 Department of Thermal Technology and Food Process Engineering, University of Life Sciences in Lublin, Głęboka 31, 20-612 Lublin, Poland; 3 Department of Biophysics, University of Life Sciences in Lublin, Akademicka 13, 20-950 Lublin, Poland; arkadiusz.matwijczuk@up.lublin.pl; 4 Laboratoire de Nutrition et Technologie Alimentaire, Institut de la Nutrition, de l’Alimentation et des Technologies Agro-Alimentaires, Université des Frères Mentouri, Constantine 25017, Algeria; ahlemlabo@yahoo.fr; 5 Departament of Lung Diseases & Rheumatology, Medical University of Lublin, 20-093 Lublin, Poland; ewa.markut-miotla@umlub.pl

**Keywords:** *Opuntia ficus indica* (L.) Mill., liquid chromatography, functional food, gluten-free pasta, antioxidant activity, phenolic acids

## Abstract

Prickly pear (*Opuntia ficus indica* (L.) Mill.) is a rich source of vitamins C, B_1_, B_2_, A, and E, minerals such as calcium, potassium, magnesium, iron, and phosphorus, as well as bioactive substances, i.e., carotenoids, betalains, and phenolic compounds. Of these, the phenolic acids, betalains, and flavonoids are notable in that they are largely responsible for the health-promoting properties of this plant. The purpose of the presented research was to first determine the antioxidant properties and the content of polyphenolic compounds (including individual phenolic acids) in prickly pear fruit, then to produce an innovative gluten-free pasta from rice-field bean flour enriched with various amounts of pear prickly fruit. The content of free phenolic acids, the sum of polyphenols and antioxidant properties of pasta were subsequently determined in the supplemented pasta. Chromatographic analysis (HPLC-ESI-MS/MS) showed a wide variety of phenolic acids. In the fruit sample, 14 acids were detected, whereas in the pasta sample without additives, 9. The dominant acid was isoferulic. The total content of free phenolic acids and the sum of polyphenols increased with increasing content of the functional additive. Moreover, the content of individual acids generally increased as the *Opuntia* fruit was added. The antioxidant activity was also positively correlated with the addition of fruit, with the content of free phenolic acids and the sum of polyphenols. Our research has shown that our innovative pasta with the addition of prickly fruit can become a source of the free phenolic acids indispensable for human health.

## 1. Introduction

*Opuntia* fig (*Opuntia ficus indica* (L.) Mill.), otherwise known as the Indian fig or cactus fig or prickly pear, belongs to the Cactus family, *Cactaceae*. These species hold high adaptability to extreme environmental conditions (drought, high temperature, UV radiation, and poor-quality soils). Originally from Mexico, it is grown in many warm climate (mainly tropical or subtropical) areas, and its fruits, flowers, and leaves are edible.

The fruits of prickly pear probably first reached Europe by way of the expedition of Christopher Columbus, hence the name ficus-indica. In countries with limited water resources, this plant is an excellent raw material for farm-based industries and has high nutritional, medicinal, and industrial value. 

Indian fig is one of the most widely cultivated cacti in the world [1,2,3]. The fruits of *Opuntia* contain a lot of water, about 93% of fresh mass, while the main components of the dry matter are sugars: glucose and fructose, as well as dietary fibers (about 50% of the dry mass). The fat content is negligible, and the protein is about 13% d. m. The fruits are also a rich source of vitamins C, B_1_, B_2_, A, and E, minerals, such as calcium, potassium, magnesium, iron, and phosphorus, as well as bioactive substances, i.e., carotenoids and phenolic compounds. Of the polyphenols, phenolic acids, anthocyanin, and flavonoid content is notable [1,4,5,6]. The peel and pulp are characterized by their held antioxidant power, due to the polyphenols, flavonoids, and betalains content [7,8]. Betalains present in the epidermis and the pulp of the prickly pear confers on it its color varying from yellow to purple (for example, betacyanins: red and blue and betaxanthines: yellow and orange). Those pigments are by-products of betalamic acid [8].

Because of their high organoleptic attractiveness and high sugar content, the fruits are eaten in both in raw and processed forms. Furthermore, the leaves (cladode) in Mexican cuisine are treated as a cooked or fired vegetable [5], and consumption generates significant reductions in serum glucose and insulin, indicating potential as a functional food candidate [9]. In addition, it should be noted that in Mexican and Chinese folk medicine, the pear pulp is used to accelerate the healing of wounds and ulcers, to treat gastrointestinal complaints, diabetes, and atherosclerosis. Studies have also confirmed its effectiveness in hyperglycemia and hypercholesterolemia. These effects are mainly associated with its held antioxidant properties [4]. The pulp also stimulates the pancreas to secrete insulin, and the fiber contained in the fruit reduces the level of LDL cholesterol. The fruit, even when eaten in small quantities, gives a feeling of fullness. This effect promotes weight reduction. Hence, the consumption of cactus pear has a positive effect on body weight and cardiovascular risk factors [10]. What is more, in unfavorable environmental conditions, Opuntia G, a substance that protects the pulp, is produced in the fruit. If applied to the skin, it increases its resistance to UV radiation, Indian fig is, therefore, utilized in anti-wrinkle formulations, stimulating skin renewal, and moisturizing [5].

Due to the high nutritional value of *Opuntia*, it is juiced [11,12,13], produced as an essential oil and a gum [14], transformed into alcohol [15], or used as a food additive [16].

Some studies report the enrichment of durum wheat pasta with 3% of *Opuntia* cladode. This serves as a source of polysaccharides, as well as phytochemical compounds, such as phenolic acids and flavonoids [17]. Indeed, Aiello et al. [17] found that pasta with 3% of *Opuntia* cladode extract is a functional food for the prevention of age-related metabolic disorders, hyperglycemia, and for maintaining normal weight.

As wheat-based pasta cannot be consumed by those with coeliac disease or gluten intolerance, gluten-free products need to be developed. As such items are usually poor nutritionally, their value must be increased through supplementation with fruits, vegetables, legumes, or herbs, ether as fresh and dried products or plant extracts.

The purpose of the presented research was, firstly, to determine the antioxidant properties and the content of polyphenolic compounds (including individual phenolic acids) in prickly pear fruit, secondly, to produce an innovative gluten-free pasta from rice-field bean flour that has been enriched with various amounts of *Opuntia* fruits. This was then analyzed to determine its content of free phenolic acids, its antioxidant properties, and the sum of its polyphenols.

## 2. Materials and Methods

### 2.1. Chemicals

Acetonitrile and formic acid requested for chromatographic analysis, ethanol for extraction and Folin-Ciocalteu reagent were purchased from J.T. Baker (Phillipsburg, NJ, USA). The used standards and DPPH (2,2-diphenyl-1-picrylhydrazyl) were provided by Sigma Aldrich (St. Louis, MO, USA). Water was purified on a MilliQ system (Millipore S.A., Molsheim, France).

### 2.2. Pasta Preparation

Fruits of *Opuntia ficus indica* (L.) Mill (yellow and orange) were brought from the local Algerian market. Fruits were washed and air dried at 40 °C overnight. The peel and seeds were separated manually from the pulp. Samples were chopped into small pieces, homogenized with an automatic press and dehydrated in a convection oven at 50 °C for 8–9 h. The dehydrated samples were ground in a hammer mill fitted with a 2 mm round orifice screen.

Gluten-free pasta was prepared on the base of rice flour and field bean flour blends at 2:1 ratio by mass (control sample). To bind together the gluten-free components, part of the rice flour was gelatinized by mixing with water 1:9 (*w*/*w*) and heated and stirred on a heating plate for 8–9 min until the temperature of 65 °C was reached. The prepared mixture was cooled down by being held for 1 h at room temperature and then kept in a fridge at 4 °C for 24 h. After storage, the gelatinized flour was seasoned by being held for 1 h at room temperature and was then added to the basic pasta recipe in an amount of 1:2 (*w*/*w*). The basic blend was supplemented with the addition of dried *Opuntia* fruits in amounts of 2.5%, 5.0%, 7.5%, 10.0%, 12.5%, and 15.0% (*w*/*w*). All the components were mixed for 15 min at 25 °C, using a KitchenAid mixer (model kPM5, St. Joseph, MI, USA) then the dough was molded and passed through the reduction rolls of a pasta machine Marcato Ampia type 150 (Campodarsego, Italy) for a uniform dough sheet and subsequently cut to the tagliatelle shape with noodles being of 5 mm width, 1.5 mm thick, and 50 mm length. Finally, the pasta samples were dried in an air oven at 40 °C for 4 h until they reached a final moisture content below 12%. The pasta was then ground and stored in sealed plastic bags at room temperature.

### 2.3. Preparation of Extracts

*Opuntia* fruit (2-gramportion), as well as supplemented pasta samples, were prepared and mixed with 40 mL of 80% ethanol. The extraction process was performed in an ultrasonic bath with a thermostat (BANDELIN electronic GmbH & Co. KG, Berlin, Germany) for 40 min at a temperature of 60 °C, ultrasound frequency of 33 kHz and a power of 320 W. The extracts were filtered, and 40 mL of 80% ethanol was added to the reminder to repeat the extraction. The obtained extractions were blended and evaporated. The dry residues were quantitatively transferred to volumetric flasks and supplemented with methanol up to 5 mL [18,19,20].

### 2.4. LC-ESI-MS/MS Analysis of Phenolic Acids

Phenolic acid content was determined according to a modified method described by Oniszczuk et al. [21]. Experiments were carried out using an Agilent 1200 Series HPLC system (Agilent Technologies, Santa Clara, CA, USA) connected to a 3200 QTRAP Mass spectrometer (AB Sciex, Redwood City, CA, USA) equipped with electrospray ionization source (ESI). Both were controlled with Analyst 1.5 software (AB Sciex, USA). This was also used for data interpretation. Separations were carried out on a Zorbax SB-C18 column (2.1 × 100 mm, 1.8-µm particle size; Agilent Technologies, Santa Clara, CA, USA) at 20 °C. The gradient method was used with mobile phases: Water with 0.1% HCOOH (A) and acetonitrile with 0.1% HCOOH (B). Herein, the injection volume was 3 µL, the flow rate was 250 µL/min and the gradient were as follows: 0–2 min: 25% B, 3–6 min: 35% B, 8–10 min: 55% B, 12–16 min: 75% B, 19–25 min: 25% B. ESI operated in the negative-ion mode at the following conditions: Capillary temperature 400 °C, curtain gas at 30 psi, nebulizer gas at 50 psi, negative ionization mode source voltage −4500 V. Triplicate injections were made for each standard solution and sample. The analytes were identified by comparing retention time and *m*/*z* values obtained by MS and MS2 with the mass spectra from corresponding standards tested under the same conditions. The identified phenolic acids were quantified based on their peak areas and by comparison with a calibration curve obtained via the corresponding standards. 

### 2.5. Determination of the Total Content of Polyphenolic Compounds

The total content of polyphenolic compounds was determined utilizing the modified Folin–Ciocalteu (FC) method [21,22]. Here, 900 μL of distilled water and 100 μL of Folin–Ciocalteu reagent were added to 100 μL of the tested extracts. The solutions were mixed and put aside. After 4 min, 1 mL of 7.7% sodium bicarbonate and 400 μL of distilled water were added. The content was mixed and placed in a water bath (40 °C) for 50 min. After this, the absorbance of the solutions was measured using a UV-VIS spectrometer (Genesys 10S UV-VIS, Thermo Scientific, Waltham, MA, USA) at a wavelength of 765 nm. The calibration curve of gallic acid was then plotted. For this purpose, 100 μL of each of the calibration solutions was collected, while 900 μL of distilled water and 100 μL of Folin–Ciocalteu reagent were added and processed following the same procedure as for the extracts above. A solution without gallic acid was used for the blank experiment. The total content of polyphenols in the tested extracts was expressed as gallic acid equivalents (GAE).

### 2.6. Radical Scavenging Assay

The antioxidative capacity of the tested extracts was measured using a 0.1 mM methanol solution of the DPPH stable radical (2,2-diphenyl-1-picrylhydrazyl). Absorbance was measured at 517 nm wavelength, and the UV-VIS spectrophotometer was calibrated to pure methanol. In the following step, absorbance of the samples containing 2.5 mL of DPPH solution and 0.5 mL of the extract was assessed. The measurements were carried out every 5 min for 30 min [22]. Based on the results, the free radical scavenging ability of the tested extracts was calculated using the following formula:

%RSA = [(*A0* − *A1*)/*A0*] × 100, (1)

where: *A0* is the absorbance of the sample except tested extracts, *A1* is the absorbance of the sample with tested extracts.

Standard antioxidant, gallic acid (0.25 mg/mL) was used as a positive control. Gallic acid proceeded by the same way as tested extracts.

### 2.7. Statistical Analysis

All the measurements were done in three replications. Results were mean values of multiple repetitions ± standard deviation (SD). Statistical analysis with ANOVA (Statistica 13.0, StatSoft Inc., Tulsa, OK, USA) was used to determine the significance of differences at α = 0.05 with Duncan’s test to evaluate the homogenous groups. Pearson’s correlation coefficients and their significance were evaluated at 0.05 and 0.01 for the tested characteristics.

## 3. Results and Discussion

### 3.1. Composition of Dried Opuntia Extracts

Polyphenol compounds are a widespread group of secondary metabolites, and many desirable biological effects depend on their presence in consumed plants. In the first stage of the study, the total content of ethanol polyphenols extracts from prickly pear fruit was examined. The technique employed to obtain extracts was a 40-min ultrasonic-assisted extraction at an elevated temperature using an 80% aqueous ethanol solution. This method was chosen because, in previously conducted experiments, this turned out to be optimal for isolating phenolic compounds from plant material [21]. In our work, the total polyphenol content was high and reached 14.948 mg GAE/g of dry matter. The results obtained are consistent with the research carried out by Yeddes et al. [7] who, when testing different species of prickly pear, revealed that this plant is rich in polyphenols. They also demonstrated the high antioxidant potential of *Opuntia* fruit extracts in DPPH test. This research is compatible with the results of Moussa-Ayoub et al. [23], who showed that the *O. ficus indica* peel and pulp demonstrated a high ability to scavenge free radicals. They explained their results by the presence of large amounts of flavonols, phenolics, as well as betacyanins in the fruit’s peel and pulp. Maataoui et al. [24] showed that the purple juice of the *O. ficus indica* has a higher antioxidant activity than the yellow-orange juice. In line with their work, in our experiment, the prickly fruit showed high potential of DPPH free radical scavenging, herein, equaling 99.78% (Table 1).

**Figure 1:**
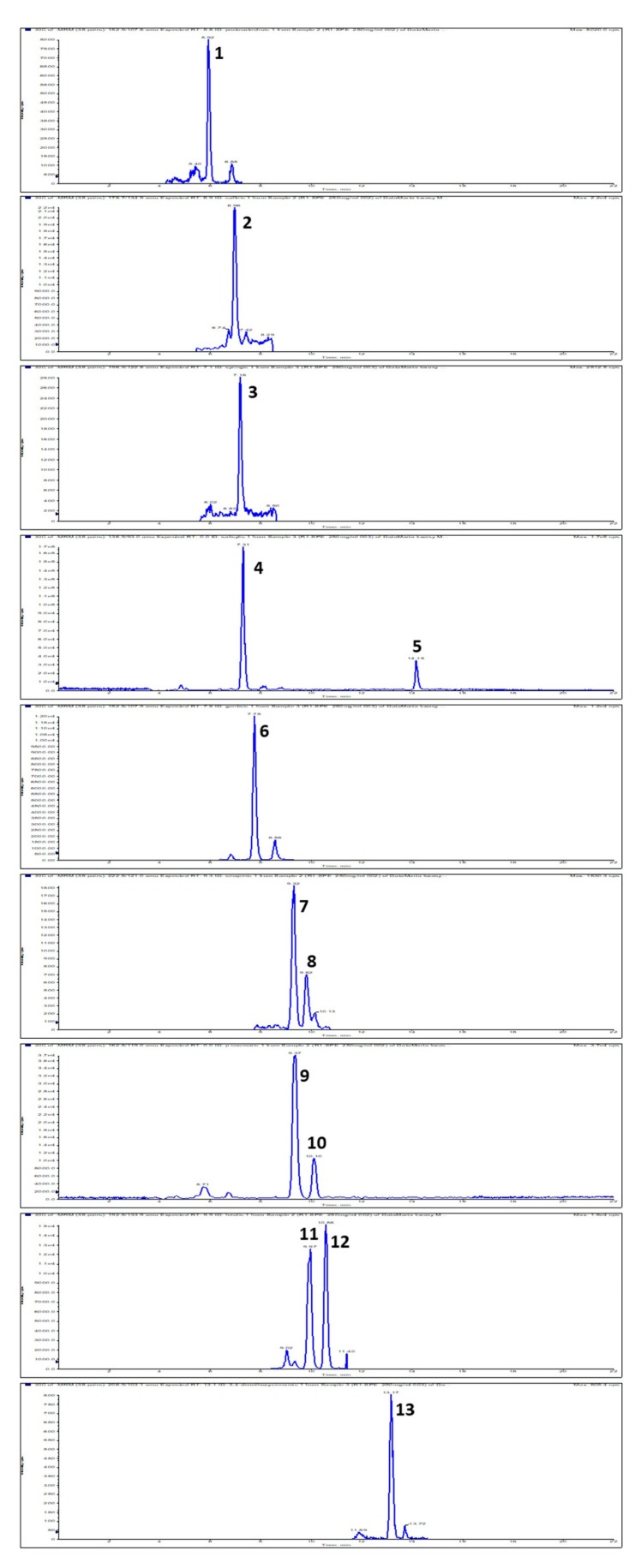
Extracted LC-MS-MRM chromatogram of phenolic acids found in prickly pear. MRM transition are given in brackets: 1, protocatechuic (m/z 152.9 → 107.8); 2, caffeic (m/z 178.7 → 134.9); 3, syringic acid (m/z 196.9 → 122.8); 4, 4-hydroxy-benzoic (m/z 136.9 → 93); 5, salicylic (m/z 136.9 → 93); 6, gentisic (m/z 152.8 → 107.9); 7, *trans*-sinapic acid (m/z 222.8 → 148.9); 8, *cis*-sinapic acid (m/z 222.8 → 148.9); 9, *p*-coumaric (m/z 162.8 → 119); 10, *m*-coumaric (m/z 162.8 → 119); 11, ferulic (m/z 192.8 → 133.9); 12, isoferulic acid (m/z m/z 192.8 → 133.9); 13, 3,4-dimethoxycinnamic acid (m/z 206.8 → 147.9).

The next step was the qualitative and quantitative analysis of the content of phenolic acids carried out by way of high-performance liquid chromatography, coupled with mass spectrometry (HPLC-ESI-MS/MS). Chromatographic analysis showed that a large variety of phenolic acids are present in the prickly fruit. Indeed, up to 14 phenolic acids were detected (Figure 1, Table 2). These were mostly benzoic acid derivatives: protocatechuic, syryngic, 4-OH-benzoic, vanilic, gentisic, salicylic, and cinnamic acid derivatives: caffeic, *trans-*sinapic and *cis*-sinapic, *p*-coumaric, ferulic, isoferulic, *m*-coumaric, and 3,4-dimetoxycinnamic. The total content of free phenolic acids reached a value of 57.957 μg/g of dry matter. Isoferulic acid, which is dominant in the prickly pear fruit, is an effective natural antioxidant in both the lipid and aquatic environment [25]. The results of the research showed that the prickly pear fruit is a great source of free phenolic acids. These are natural antioxidants, and the consumption of foods with a high content of phenolic acids has important health benefits due to their anti-cancer, anti-bacterial, and anti-inflammatory properties.

Because of the high antioxidant potential and high polyphenolic compound content, the authors designed a gluten-free food product enriched with different percentages of prickly pear. In this way, pasta was created, the basic recipe of which was rice flour and field bean flour blend in the amount of 2:1. As an addition to the basic recipe, dried *Opuntia* fruit was used in the amount of 2.5%, 5.0%, 7.5%, 10.0%, 12%, and 15.0%. The pasta was subsequently tested for the antioxidant properties and the polyphenols content.

### 3.2. Results of Analysis of Extracts from Pasta Enriched with Different Percentages of Opuntia Fruits 

In the assessment of the total content of polyphenols in the pasta blends, the results clearly showed that the polyphenol content increased along with the increase of prickly pear fruit content. Herein, the sum of polyphenols reached 4.641, 4.983, 5.447, 5.672, 6.068, and 6.367 and 6.528 mg GAE/g dry matter for the control and enriched samples at 2.5%, 5.0%, 7.5%, 10.0%, 12.5%, and 15.0% of prickly pear fruit, respectively.

On establishing the antiradical scavenging ability of pasta supplemented with *Opuntia* fruit, the antioxidant properties of the tested pasta in relation to the DPPH free radical were also high. The percentage of DPPH scavenging was 83.02, 93.95, 94.52, 95.16, 95.76, 96.23, and 97.04 for samples containing 0%, 2.5%, 5.0%, 7.5%, 10.0%, 12.5%, and 15.0% of *Opuntia* fruit, respectively (Table 1). The test confirmed that the increased amount of prickly pear in the pasta, enhanced concurrently their antioxidant properties. Thus, we observed that an increase in the amount of functional additive brought about an increase in polyphenol content and an increase in antioxidant properties. The scavenging capacity against DPPH radicals was positively correlated with total polyphenols content and with the free phenolic acids content at α=0.05 (Table 3). 

The maximum scavenging ability of free radicals by all extracts was obtained after 10 min. As previously reported by Kuti [26] and Gentile et al. [27], the beneficial effect on the human body brought about by consumption of prickly pear is largely due to their antioxidant properties resulting from the content of polyphenol compounds. Moreover, the content of individual phenolic acids increased as the amount of prickly fruit increased. The exception was salicylic acid, the content of which steadily decreased and reached the lowest value in the sample enriched with 15.0% of the additive. This is conditioned by the fact that the basis pasta, i.e., a mixture of rice and field bean flour, is richer in this acid than the prickly pear fruit. It is, therefore, understandable that as the dried *Opuntia* was added, the amount of this component was reduced. Furthermore, the content of *m*-coumaric acid remained the same in all samples. 

Phenolic compounds are secondary metabolites in plants so they can be variously distributed in the structure of plants, e.g., insoluble phenolics in cell walls, but soluble phenols within vacuoles of vegetable cells. Therefore, in various parts of plants (fruits, flowers, leaves) there can be different distribution and concentration of phenolic compounds [28]. Moreover, variations in phenol concentration during food processing or drying may be due to the early degradation of phenolic compounds favored by high temperatures and prolonged exposure to heat due to the release of phenolic substances caused by the rupture of the ether, ester, or acetal covalent bonds [29]. Thermal degradation may occur at the same time that the phenolic substances are released, but its concentration may be observed when maximal degradation is reached at the temperatures evaluated [30]. It is especially visible at high-temperature treatment, as reported by Mutari et al. [31], who suggest that higher temperatures can improve the solubility of phenolic compounds leading to the breakdown of cellular structures and improving the release of phenolic compounds, such phenolic acids (ferulic, galic, and vanillic acid), previously bound to the macromolecules of the cell wall. Moreover, processing can accelerate the release of phenolic compounds due to the breaking of cellular constituents. Drying in low temperature (40 °C and 50 °C in our experiment) is not sufficiently high to deactivate hydrolytic or oxidative enzymes which may have an influence on phenolic content in tested materials [28]. Therefore, it was not possible to prevent loss of phenolic compounds after drying.

The total content of free phenolic acids also increased with the increase of the functional additive content in the pasta samples, reaching values of 26.671, 28.359, 29.525, 32.503, 34.097, 36.153, and 38.876 μg/g of dry matter, respectively, for the control without addition and samples enriched with the addition of 2.5%, 5.0%, 7.5%, 10.0%, 12.5%, and 15.0% of fruit. 

The obtained test results thus showed that the antioxidant activity of pasta was positively correlated with the amount of added prickly pear fruit, and the content of free phenolic acids and the sum of polyphenols. Pearson’s coefficients, indicating the relationship between functional additive, polyphenols, free phenolic acid content and antioxidant activity (measured after 10 min), are presented in Table 3. Herein, it can be seen that very high positive correlations were established between the applied fruit addition and the polyphenol content (r = 0.994) and between the amount of enrichment additive and the content of free phenolic acids (r = 0.995). Of note, previous research has shown that aglycones have a higher antioxidant activity than glycosidic forms or those connected by different types of bonds [25,32]. The antioxidant activity of polyphenols is also dependent on the number of hydroxyl groups in the molecule and can be enhanced by spherical effects, as well as by synergistic and antagonistic interactions of the compounds present in the matrix and in the extracts. Thus, an increase in the content of polyphenolic compounds entails an increase in antioxidant properties.

## 4. Conclusions

The presented study indicates that *Opuntia* fruit (prickly pear) is a rich source of phenolic compounds, especially the benzoic acid derivatives: protocatechuic, syryngic, 4-OH-benzoic, vanilic, gentisic, salicylic, as well as the cinnamic acid derivatives: caffeic, *trans*-sinapic, and *cis*-sinapic, *p*-coumaric, ferulic, isoferulic, *m*-coumaric and 3,4-dimetoxycinnamic. Due to their high antioxidant potential, such fruit can be used as a valuable food additive. In our study, a gluten-free pasta based on a rice-field bean blend was prepared with the addition of 2.5–15.0% of prickly pear fruits. This fortified pasta showed high content of phenolic compounds, especially at levels of 12.5% and 15.0% of the additive. Herein, the maximum scavenging ability of DPPH free radical by all pasta extracts was obtained after 10 min and the increased amount of functional additive brought about an increase in antioxidant properties. Extracts from pasta with the largest (15.0%) addition of prickly fruit showed the greatest antioxidant properties. Our research has demonstrated that innovative gluten-free pasta supplemented with *Opuntia* fruits is a good source of natural antioxidants, and thus, can improve the quality of health and life of coeliac consumers. In the future, products such as this can enrich the range of gluten-free products that have entered the ranks of functional foods.

## Figures and Tables

**Table 1:** Radical scavenging activity of gluten-free pasta samples depend on time and *Opuntia* fruit addition (n = 3 mean ± SD).

Radical Scavenging towards DPPH (%)											
Time (min)	Addition of *Opuntia* (%)										
	0	2.5	5.0	7.5	10.0		12.5		15.0	*Opuntia* Fruit	Gallic Acid
0	18.25 ^a^	13.57 ^b^	14.69 ^b^	17.92 ^b,c^		22.18 ^c^		33.37 ^d^	33.37 ^d^	35.38 ^e^	99.37 ^c^
	± 0.22	± 0.05	± 0.10	± 0.06		± 0.68		± 0.89	± 0.01	± 0.01	± 0.01
5	82.75 ^a^	93.95 ^b^	93.95 ^b^	94.67 ^b,c^		94.26 ^b,c^		96.03 ^c^	94.92 ^b,c^	96.88 ^c^	100.00 ^b,c^
	± 0.33	± 1.32	± 1.21	± 0.59		± 1.32		± 0.01	± 0.04	± 0.56	± 0.00
10	83.02 ^a^	93.95 ^b^	94.52 ^b,c^	95.16 ^c^	95.76 ^c^		96.23 ^c,d^		97.04 ^d^	99.78 ^e^	100.00 ^c^
	± 0.01	± 0.67	± 0.11	± 0.21	± 0.03		± 0.02		± 0.33	± 0.23	± 0.00
15	83.02 ^a^	93.95 ^b^	94.52 ^b,c^	95.16 ^c^	95.76 ^c^		96.23 ^c,d^		97.04 ^d^	99.78 ^e^	100.00 ^b,c^
	± 0.02	± 0.01	± 0.00	± 0.03	± 0.00		± 0.03		± 0.00	± 0.03	± 0.00
20	83.02 ^a^	93.95 ^b^	94.52 ^b,c^	95.16 ^c^	95.76 ^c^		96.23 ^c,d^		97.04 ^d^	99.78 ^e^	100.00 ^b,c^
	± 0.00	± 0.01	± 0.02	± 0.01	± 0.03		± 0.01		± 0.00	± 0.00	± 0.00

^a–e^ similar letters in rows indicate insignificant differences at α=0.05.

**Table 2:** Content of phenolic acids in *Opuntia* fruit and gluten-free pasta samples enriched with *Opuntia* fruit addition (n = 3; mean ± SD).

Addition of *Opuntia*(%)		Content of Phenolic Acids (µg/g)													
	Protocatechuic	Caffeic	Syryngic	4-OH-Benzoic	Vanilic	Gentisic	*trans*- Sinapic	*cis*- Sinapic	*p*-Coumaric	Ferulic	Isoferulic	*m*-Coumaric	3,4-Dimetoxycinnamic	Salicylic	Sum
*Opuntia* fruit	0.616^d^ ± 0.008	0.404	1.552	3.558^d^	BQL	0.196^d^	1.025	0.042	1.680^d^	4.160^e^	43.223^e^	0.252^ab^	0.724^d^	0.515^a^	**57.957**
		± 0.002	± 0.043	± 0.038		± 0.005	± 0.003	± 0.0001	± 0.011	± 0.105	± 0.345	± 0.001	± 0.008	± 0.009	
0	0.087^a^ ± 0.002	ND	ND	1.046^a^	ND	0.035^a^	ND	ND	0.252^a^	0.393^a^	23.124^a^	0.268^b^	0.252^a^	1.214^d^	**26.671**
				± 0.019		± 0.0002			± 0.008	± 0.013	± 0.2052	± 0.001	± 0.014	± 0.022	
2.5	0.093^a^ ± 0.003	ND	ND	1.492^ab^	ND	0.040^ab^	ND	ND	0.278^a^	0.408^a^	24.416^ab^	0.247^a^	0.265^a^	1.120^cd^	**28.359**
				± 0.043		± 0.0000			± 0.003	± 0.008	± 0.012	± 0.012	± 0.005	± 0.021	
5.0	0.112^ab^ ± 0.003	ND	ND	1.612^b^	ND	0.046^b^	ND	ND	0.307^ab^	0.409^a^	25.636^b^	0.258^ab^	0.277^ab^	0.869^c^	**29.525**
				± 0.051		± 0.0000			± 0.001	± 0.012	± 0.018	± 0.012	± 0.010	± 0.031	
7.5	0.184^b^ ± 0.004	ND	ND	2.316^bc^	ND	0.051^b^	ND	ND	0.358^b^	0.557^ab^	27.677^bc^	0.240^a^	0.292^b^	0.828^bc^	**32.503**
				± 0.033		± 0.0001			± 0.012	± 0.014	± 0.122	± 0.011	± 0.001	± 0.029	
10	0.192^b^ ± 0.006	ND	ND	2.644^c^	ND	0.057^bc^	ND	ND	0.420^bc^	0.735^b^	28.751^c^	0.266^b^	0.316^b^	0.716^b^	**34.097**
				± 0.024		± 0.0002			± 0.024	± 0.032	± 0.030	± 0.007	± 0.000	± 0.001	
12.5	0.242^bc^ ± 0.007	ND	ND	2.720^c^	ND	0.061^c^	ND	ND	0.486^bc^	0.992^c^	30.428^cd^	0.246^a^	0.336^bc^	0.642^ab^	**36.153**
				± 0.038		± 0.0003			± 0.007	± 0.027	± 0.424	± 0.006	± 0.008	± 0.018	
15.0	0.344^c^ ± 0.005	ND	ND	2.856^c^	ND	0.063^c^	ND	ND	0.536^c^	1.544^d^	32.321^d^	0.258^ab^	0.362^c^	0.638^ab^	**38.876**
				± 0.132		± 0.0001			± 0.006	± 0.065	± 0.692	± 0.006	± 0.006	± 0.013	

^a–e^ similar letters in columns indicate insignificant differences at α = 0.05.

**Table 3:** Pearson’s correlation coefficients for gluten-free pasta supplemented with *Opuntia* fruit addition.

	Total Polyphenols	Free Phenolic Acid	DPPH Radical Scavenging Activity
*Opuntia* fruit content	0.994 **	0.995 **	0.768 *
Total polyphenols		0.979 **	0.795 *
Free phenolic acids			0.729 *

* significant at α = 0.05, ** significant at α = 0.01.
